# A structured literature review of the health infodemic on social media in Africa

**DOI:** 10.4102/jamba.v15i1.1484

**Published:** 2023-09-29

**Authors:** Charity Hove, Liezel Cilliers

**Affiliations:** 1 Department of Information Systems, Faculty of Management and Commerce, University of Fort Hare, East London, South Africa

**Keywords:** infodemic, social media, COVID-19, risk communication, Africa, misinformation, COVID-19 pandemic

## Abstract

Infodemics is a multi-faceted threat that needs to be dealt with by governments during public health emergencies. This strategic review described the role of social media platforms in creating and reinforcing an infodemic during health pandemics in Africa. The inclusion criteria for the review were African research on infodemics on social media during pandemics, epidemics or endemics in the past 10 years. A structured literature review, making use of the Sample, Phenomenon of Interest, Design, Evaluation, Research (SPIDER) scoping review methodology framework, identified scholarly publications from various academic databases. A total of 41 articles met the eligibility criteria. The six factors identified included stakeholders, socio-economic context, conspiracy theories, sources of information, government responses and verification mechanisms. The findings of this study indicate that governments needs to include infodemics in the risk communication strategy for public health emergencies. Verification of misinformation can mitigate the effects of conspiracy theories while the socio-economic context of the audience will influence planning strategies to mitigate infodemics on social media.

**Contribution:**

This study contributes to the knowledge base of risk communication during pandemics in Africa by providing a review of how infodemics on social media have influenced the COVID-19 pandemic on the continent. The results also provide a foundation for the research agenda in this research field that will provide an evidence-based response to the pandemic in Africa.

## Introduction

The emergence of SARS-CoV-2 and the corresponding disease of coronavirus disease 2019 (COVID-19) took the global community by storm in early 2020. The virus originated in Wuhan China and was declared a pandemic by the World Health Organization (WHO) on 11 March 2020 (WHO 2020). The WHO advised governments worldwide that social media platforms should be included in their risk communication strategy for public health emergencies or natural hazards. The benefit of social media platforms as risk communication tools are the two-way communication between government and citizens that can provide accurate information to a large audience and collect data in real-time from emergency areas to allocate resources more efficiently (Amani et al. 2020).

The term ‘infodemics’ means the spread of an excessive amount of information during a health crisis (Adebisi, Rabe & Lucero-Prisno 2021b). While the term has been used during previous public health emergencies, the sheer magnitude of information on social media about COVID-19 propelled this research area to the forefront since 2020. The dramatic increase in social media users during the COVID-19 pandemic also brought about the risk of misinformation that can be created, shared and consumed easily by a worldwide audience (Katurura & Cilliers 2018).

Health misinformation is defined as ‘information that counters the best available evidence from medical experts at the time’ (Singh et al. 2020:1). A recent study conducted revealed that 19% of citizens in Africa believed that the pandemic was designed to reduce the world’s population, 22% thought the ability to seize your breath for 10 s meant that you do not have COVID-19 and 14% thought that COVID-19 had minimal effect on the black population (Adebisi et al. 2021b). Misinformation undermines health institutions’ efforts to communicate accurate information as resources need to be allocated to deal with the misinformation first (Katurura & Cilliers 2018).

The WHO Director General, Tedros Adhanom Ghebreyesus, ascertained the seriousness of misinformation during a pandemic when he declared that the world was not just fighting a COVID-19 pandemic, but the infodemic that came along with it (Zarocostas 2020). In essence, this implies that the COVID-19 infodemic is equivalent to a pandemic on its own – a pandemic within a pandemic.

## Aim of the research

Research about the infodemic on social media following COVID-19, is still in its infancy. The research that has been conducted on the topic of information on epidemics shared on social media platforms during a public health crisis in Africa lacks a regimented and carefully iterative analysis of topics (WHO 2020; Zarocostas 2020). As a result, the purpose of this strategic review of recently published and relevant literature was to describe the available research concerning the role of social media platforms in creating and reinforcing an infodemic during health pandemics in Africa. To provide a more robust discussion of the subject matter, information regarding other viral epidemics such as human immunodeficiency virus (HIV), Monkeypox and Ebola virus may also be drawn upon in the structured literature review.

## Literature review

An infodemic is not modern phenomenon during public health crises. Historically, there has always been misinformation and rumours associated with health crises (Santos-D’Amorim & De Oliveira Miranda 2021). The origins of infodemics can be traced back to Rothkopf (2003) who wrote an article about the information epidemic during the severe acute respiratory syndrome (SARS) epidemic in 2003. The term infodemic is a combination of the word’s ‘information’ and ‘epidemic’. Infodemics is also linked to infodemiology, which is defined as the ‘science of distribution and determinants of information in an electronic medium, specifically the internet, or in a population, with the ultimate aim to inform public health and public policy’ (Eysenbach 2009:2257).

Social media provides a convenient channel for the spread of information during pandemics as it is easily accessible and can reach a wide audience (Jolly et al. 2020). Using social media as a risk communication tool means that the barriers to public involvement in emergency responses are lowered. Simultaneously, the government can respond more effectively to the emergency as they can monitor new information for situational awareness during the event (Diaz et al. 2020). During a pandemic, people use location-based social network services, such as Twitter or Facebook, that collect time-stamped and geo-located data for authorities that provide information about their environment in real time. The popularity of social media platforms has meant a sharp increase in their usage during the COVID-19 pandemic, as shown by the 45% increase in Twitter usage during the first 3 months of 2020 (Jolly et al. 2020).

One of the disadvantages of using social media to distribute information during a public health crisis is that there is little validation of the information that is posted on social media when authors can create and share content that is characterised by opinion or hearsay. False information can be spread around the world in a matter of seconds. There are two categories of false information as identified in the literature. The first category is disinformation, which is defined as information being fabricated intentionally for the purpose of creating rumours. The second category, misinformation, refers to inaccurate information that is unintentionally created or shared with a wider audience (Santos-D’Amorim & De Oliveira Miranda 2021). Both disinformation and misinformation have a negative impact on public health efforts as the information undermines the efforts of government or health institutions to fight the pandemic, leading to mistrust among the public because of the negative perception’s misinformation creates (WHO 2020, 2021a).

## Research methods and design

The research methodology for this study comprised a systematic review of the literature in order to identify the current knowledge gap in the research field. The purpose of this strategic review of recently published and relevant literature was to describe the available research concerning the role of social media platforms in creating and reinforcing an infodemic during health pandemics in Africa.

### Identify relevant studies

This review followed the Preferred Reporting Items for Systematic Reviews and Meta Analyses Extension for Scoping Reviews (PRISMA-ScR) checklist and reporting guideline (Tricco et al. 2018). The research made use of the Sample, Phenomenon of Interest, Design, Evaluation, Research type (SPIDER) methodology for selecting relevant articles in order to achieve relevant qualitative outcomes. The Phenomenon of Interest was infodemics on social media during public health crises (including pandemics, epidemics and endemics) while the sample included studies conducted or focusing on Africa. The design, evaluation and research type parameters were broad, including empirical studies, literature reviews and theoretical articles as the research field is still in its infancy and enough studies needed to be included to produce a worthwhile and meaningful contribution to the knowledge area. A scoping review protocol was developed; however, it was not registered or published in any journal prior to the start of this review. On request, the protocol will be made available.

The literature search that informed this study was carried out systematically using Google Scholar, Science Direct, PubMed, Scopus and ProQuest. The search blocks used included: (1) *COVID-19 infodemic + Africa, Zika virus infodemic + Africa,* (2) *Ebola infodemic + Africa, HIV/AIDS infodemic + Africa,* and (3) *Monkeypox infodemic + Africa*. Articles published in English between January 2010 and July 2022 were included in the study.

### Inclusion and exclusion criteria

The relevance of articles was determined based on the inclusion criteria of addressing misinformation, addressing the African context in a meaningful way and infodemics on social media during public health crises. The exclusion criteria were based on the following: (1) articles that were not focusing on Africa during a public health emergency; (2) articles written by organisations, for example, WHO, UNICEF, etc. – only academic research articles with research methodologies were included; (3) articles that did not focus on infodemics or any identified relevant topic such as misinformation; (4) articles without full access; (5) articles that were not in English and (6) duplicate articles.

### Study selection

Following the search process, the article title and abstract were examined for relevance to the study by the principal investigator (CH), who exported the results into an Endnote library. Duplicate articles were removed after which the principal investigator (CH) and co-screener (LC) analysed the articles. In cases where the abstract indicated continued relevance, the full text of the article was retrieved and included in the review. The inclusion criteria resulted in the selection of 41 articles that were ultimately included in the review.

### Data extraction

**FIGURE 1:**
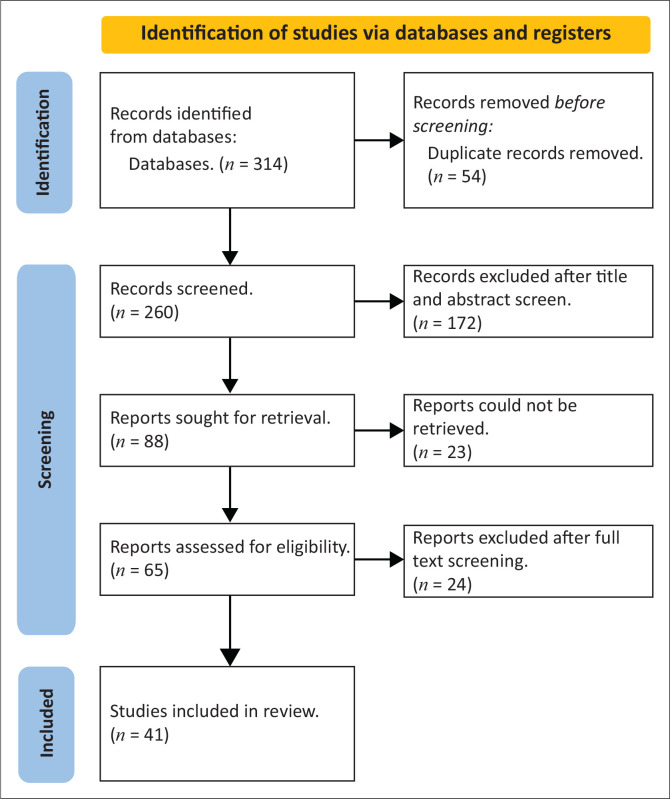
Adapted preferred reporting items for systematic reviews and meta-analyses flow chart, demonstrating search and selection of studies.

Thematic analysis was conducted on the articles to identify key themes characterising the literature by the two researchers independently of each other (CH and LC). Where discrepancies were found, the researchers came to a consensus before a decision was made about the article. The location, methodologies, pandemic, categories measured and findings of the studies were all described. Figure 1 provides the oversight of how the articles were excluded while Table 1-A1 provides a summary of the articles included in the study.

### Ethical considerations

This study is part of a postdoctoral research project and the researchers sought research ethical clearance. The clearance was granted under number HOV001-22 from the University of Fort Hare’s Inter-Faculty Research Ethics Committee (IFREC).

## Discussion of results

The research area of infodemics is still growing although the term was first introduced in 2003 with the SARS pandemic. In this structured literature review, the 41 articles listed in the appendices were included in the analysis to support the content analysis and discussion in this section. These articles were all written in the past 4 years, with one article published in 2019, 10 articles published in 2020, 22 articles published in 2021 and 8 articles published in 2022. Fifty-four percent of the articles were published in 2021 as a response to the COVID-19 pandemic, which was at its height the previous year. Many African countries imposed a lockdown, closed schools and implemented social distancing to restrict infections, while a subsequent increase in social media usage by African citizens provided new research agendas and opportunities in the field of infodemics. Not surprisingly, the majority of the articles (85%) dealt with infodemics in the context of COVID-19, while five articles drew parallels between the Ebola, HIV and COVID-19 pandemics. Only one article, which was written in 2019, dealt exclusively with misinformation during the Ebola crisis (Balami & Meleh 2019).

The most prevalent research methodology, as reported in 27 articles, was a qualitative research approach, followed by a quantitative research approach with 12 articles, and lastly, 2 articles made use of a mixed methods approach. Qualitative research is used to provide in-depth insight into research questions when the study area is not well known, as is the case with infodemics, or if there are unstructured data that need to be analysed (Bryman 2016).

While some of the articles investigated social media and infodemics in general, there were specific social media platforms discussed in the study ranging from Facebook, Twitter, direct messaging systems such as WhatsApp or Facebook Messenger and video calling services for example, Zoom. This shows that misinformation can be shared on either social media platforms or networks.

### Social media infodemic

There is a common misperception that social media is the main channel responsible for the infodemic during COVID-19 (Adekoya & Fasae 2021; Demuyakor, Nyatuame & Obiri 2021; Ennab et al. 2022:20; Lucas et al. 2022; Madziva et al. 2022; Okereke et al. 2021;Osuagwu et al. 2021; Shobowale 2021; Stewart et al. 2022). This misperception occurred because of the original infodemiology study that was conducted by Eysenbach in 2009, which portrayed social media as a way of creating and enabling infodemics to flourish. While many are quick to associate social media with infodemics, social media is not the only channel that can be used to spread an infodemic. Traditional media channels are equally responsible for the spread of infodemics because of the large amount of information distributed during public health crises (Lucas et al. 2022). However, the difference between content created on social media as opposed to traditional media is that journalists need to adhere to a specific code of ethics when producing content that includes verification of the facts, whereas citizens can create and share information on social media without any verification of the data. Social media usage in Africa has increased exponentially since COVID-19 while many African countries imposed a lockdown to curb the spread of the disease. These measures contributed to more content being created, exchanged and consumed, which supported the infodemic during the COVID-19 infodemic (Adekoya & Fasae 2021; Santos-D’Amorim & De Oliveira Miranda 2021).

While the majority of the articles included in the study focused on COVID-19, five articles combine a discussion of COVID-19 and other epidemics that occurred in Africa (Shobowale 2021). This finding may signify the view that infodemics have started with COVID-19. However, this is not true as the term was used during the SARS pandemic in 2003 (Ijab, Shahril & Hamid 2021).

Fake news is hard to fight not only because it can spread quickly on social media, but there is often a political and financial motive to share such information (Adebisi et al. 2021b; Ademola et al. 2021). Since the start of the COVID-19 pandemic, there has been an increase in scams related to treatment, medication, testing and vaccines on social media. The importance of this public health threat becomes clear when one considers the correlation between an infodemic and COVID-19-related stress, suggesting that the infodemic is dangerous to one’s health and could affect healthy living in the long run (Shobowale 2021). Fake news often increases public anxiety, anger and frustration around these topics, that may lead to poor compliance by the public to protect themselves from COVID-19 (Ademole et al. 2021).

Social media platforms have responded to the threat of an infodemic and fake news by putting in place safeguards to make it easier to recognise fake news (Shobowale 2021). However, there are concerns if social media sites can be trusted to make these changes if it will affect their profitability (Zenone et al. 2023). Governments and public health agencies need to confront misinformation on social media and put in place policies and mechanisms to deal with the issue and bring those who are responsible for creating and spreading misinformation to justice (Ademole et al. 2021). Actions such as maintaining an official social media presence, making use of public opinion leaders in the fight against misinformation, and creating tools to facilitate the easy spread of accurate information will have a far-reaching effect to mitigate misinformation on social media (Shobowale 2021).

While governments need to guard against misinformation, they also have a responsibility to share information with each other. The COVID-19 pandemic has highlighted the need to reorganise data and streamline data sharing at all levels of government. They are further needed to share health information systems data, including observational studies and disease monitoring and surveillance programmes to ensure that all countries have the necessary information to implement rapid protocols and strategic plans for potential pandemics such as COVID-19 (Lal et al. 2022).

### Socio-economic context

Madziva et al. (2022) stated that the notion that information shared on social media is a ‘magic bullet’ that will solve the intended problem when the audience receives the message. The authors suggest that not all members of the audience will interpret the message in the same way. Information shared on social media must consider the demography, culture, religion, health literacy and socio-economic conditions of the intended audience (Adebisi et al. 2021b; Okereke et al. 2021).

Rural communities have little or no access to accurate health information because of the unavailability of the Internet or even traditional media. The lack of health information often leads to a low level of health literacy in the rural areas of low- and middle-income countries. Health literacy allows an individual to understand abstract concepts such as germ theory, infectivity and prophylaxis such as vaccines. When health information is available but ambiguous or not accurate, the result is doubt and hostility from the citizens and mistrust in health authorities (Okereke et al. 2021).

### Conspiracy theories on social media

Social media is the biggest source of the COVID-19 infodemic and related conspiracy theories (Demuyakor et al. 2021; Ufearoh 2020). The biggest culprit in this regard was Facebook, which accounted for 52.3% of vaccine infodemic when compared with WhatsApp, Twitter and YouTube (Demuyakor et al. 2021). Stewart et al. (2022) identified five potential domains of impact where misinformation can cause potential harm (Table 1).

**TABLE 1:** Misinformation domains.

Domain of impact - harms	Consequence
Physical	Limited accurate knowledge about available treatments. Misplaced actions
Social	Victimisation and stigma.
Economic	Falling for scams. Panic buying.
Political	Limited trust in officials. Rejection of official guidelines. Disregard of government-led responses.
Psychological	Mental health epidemic. Extreme anxiety. Long-term depression.

Source: Stewart, R., Madonsela, A., Tshabalala, N., Etale, L. & Theunissen, N., 2022, ‘The importance of social media users’ responses in tackling digital COVID-19 misinformation in Africa’, *Digital Health* 8, 20552076221085070. https://doi.org/10.1177/20552076221085070.

Gagliardone et al. (2021) warns that conspiracies on social media are often interpreted as either falsehood versus fact; centre versus periphery dichotomy or an over-reliance on datasets that can be associated with moral panic. This debate on conspiracy theories is open to misuse where opportunistic individuals make use of the chance to include government corruption and political opposition in the conversation (Ogola 2020). These types of accusations normally take place around the periphery of the discussion and are used to influence those that do not have a strong opinion on the matter. This is true of the anti-vaccination movement that used the COVID-19 agenda to broadcast their message that one should not only be suspicious of new vaccines but any vaccine (Ajekwe 2022). The 5G conspiracy in Nigeria and Zimbabwe was linked to the government’s corruption and ill-will towards the citizens of the country, while in South Africa this particular conspiracy marginally intersected with local political debates. There is also the existential response to conspiracy theories, such as the Bill Gates conspiracy, where broader suspicion of foreign or Western interference in African affairs is incorporated into the narrative (Mare & Munoriyarwa 2022).

Africa Check reports that the main categories of misinformation focus on the cause of the virus, how to prevent infection, treatment and alleged sinister objectives of superpowers and wealthy businessmen (Mare & Munoriyarwa 2022). Table 2 provides an overview of the most popular conspiracy theories found in the literature.

**TABLE 2:** Analysis of conspiracy theories across Africa.

Conspiracy theory	Countries	Source
5G as the origin or method of spreading the virus and tracking people via microchips	Nigeria, South Africa, Zimbabwe	Madziva et al. (2022), Lucas et al. (2022), Gagliardone et al. (2021), Mare and Munoriyarwa (2022)
Bill Gates conspiracy	South Africa, Nigeria	Madziva et al. (2022), Lucas et al. (2022), Gagliardone et al. (2021), Mare and Munoriyarwa (2022)
COVID-19 is a Chinese biological weapon	Nigeria, Malawi	Madziva et al. (2022), Lucas et al. (2022), Olatunji et al. (2020), Manda (2020)
Religious considerations	Nigeria, Kenya, Cameroon, Ghana, Tanzania, South Africa, and Uganda	Madziva et al. (2022), Lucas et al. (2022), Adebisi et al. (2021b), Olatunji et al. (2020), Schmidt et al. (2020)
Population control strategy	Nigeria, Sub-Saharan Africa, Kenya, Cameroon, Ghana, Tanzania, South Africa, and Uganda	Osuagwu et al. (2021), Adebisi et al. (2021b: 28), Olatunji et al. (2020:36)
Government and media exaggeration	Nigeria, Sudan, Kenya, Malawi, Cameroon, Ghana, Tanzania, South Africa, and Uganda	Okereke et al. (2021), Adebisi et al. (2021b), Ufearoh (2020), Olatunji et al. (2020), Manda (2020), Schmidt et al. (2020), Aiyebelehin and Mesagan (2021)
Unproven methods to prevent contracting COVID-19 (alcohol, high temperatures, higher socio-economic status, sea lettuce or salt, disinfectant, alkaline foods, vitamin D or C, steam with herbs, hydroxychloroquine)	Tanzania, Kenya, Nigeria, Zimbabwe, Sub-Saharan Africa, Kenya, Cameroon, Ghana, South Africa, and Uganda	Balami and Meleh (2019), Shobowale (2021), Ennab et al. (2022), Osuagwu et al. (2021), Lucas et al. (2022), Okereke et al. (2021), Adebisi et al. (2021b), Ufearoh (2020), Ajekwe (2022), Mare and Munoriyarwa (2022), Schmidt et al. (2020), Kunguma (2021), Aiyebelehin and Mesagan (2021), Aduloju (2021), Atuguba and Atuguba (2020)

*Note:* Please see the full reference list of the article, Hove, C. & Cilliers, L., 2023, ‘A structured literature review of the health infodemic on social media in Africa’, *Jàmbá: Journal of Disaster Risk Studies* 15(1), a1484. https://doi.org/10.4102/jamba.v15i1.1484, for more information.

COVID-19, coronavirus disease 2019.

There are strategies that have been developed to mitigate the potential and spread of misinformation on social media. These include (Wasserman et al. 2021):

- Providing credible, accurate information as an alternative to misinformation.
- Encouraging self-efficacy to detect misinformation by raising awareness of how to recognise and identify misinformation on social media.
- Criminalising misinformation with severe consequences.
- Using infoveillance on social media sites to increase the early detection of misinformation.
- Implementing technical approaches to identify misinformation such as the ‘fake tweet generator’ and the reverse image search tool.
- Debunking misinformation on the same platform where it has been found.
- Involving social media companies to fight misinformation for example, by deleting accounts created with the intent of spreading conspiracy theories.

### Source of information

The findings indicate that scholars identified different sources of information during a health infodemic. These are discussed in the following subheadings:

#### Traditional media

News consumption increased during the COVID-19 pandemic, hence sources such as print and electronic media need to provide accurate information (Mare & Munoriyarwa 2022). During the recent pandemic, radio, television, friends and social media were found to be the sources of information most cited, while newspapers and banners were not mentioned. This is probably because of the national lockdown that meant citizens’ movement was restricted and they could not access newspapers or travel to see banners (Manda 2021). Information sources such as the WHO, the America National Centre of Disease Control, credible academic institutions and national healthcare organisations were seen as trustworthy and inspiring confidence when COVID-19 information is reported in traditional media (Demuyakor et al. 2021; Olatunji et al. 2021).

Wasserman et al. (2021) reported that the content analysis of 681 front-page news stories across 11 English-language publications in South Africa found that almost half of the stories used an alarmist narrative while more than half of the stories had a negative tone. Newspaper stories focused on the impact of the COVID-19 pandemic in a sensationalism manner that could spread panic and did not provide enough information to empower citizens.

#### Word of mouth

Another theme that was identified from the articles suggests that in rural Africa, there is the existence of localised health misinformation (Demuyakor et al. 2021; Shobowale 2021). In rural Africa, health misinformation occurs via word of mouth (WOM) in areas where social media access is either non-existent or limited (Manda 2021). The WOM is an offline channel for spreading information and is characterised by hearsay, rumours and misinformation. The WOM can occur among community members that share health information, but another source is religious leaders in the community who have chosen to frame the COVID-19 pandemic in a spiritual interpretation. Ogola (2020) found that religious scholars in Nigeria claimed that Muslims were immune from COVID-19, while others claimed that the virus can be stopped by prayer, and social distancing was not necessary. The challenge WOM poses is that it is a conduit for misinformation that is not addressed by policies fighting misinformation because most policies mainly focus on online misinformation. This is an inherent bias that public health institutions should address to mitigate infodemics on traditional and social media communication channels.

#### Social media

During lockdown period, citizens used social media to obtain accurate information about the status of the pandemic, announcements from the government or other trusted sources, and measures to take to prevent or treat the infection (Adekoya & Fasae 2021). The most used social media channel during the COVID-19 pandemic was WhatsApp, followed by Zoom, Facebook and YouTube (Adekoya & Fasae 2021; Chimoyi et al. 2021). Government can make use of social media platforms to raise public awareness about public health topics. However, these public health campaigns should be tailored to the social media platform and intended audience (Dzinamarira et al. 2021). A human-centred design approach with specific objectives, as opposed to uncoordinated messages on social media, was found to yield the best results to influence behavioural change. An additional consideration when using social media to deliver public health information is the high data cost associated with streaming video content. An offline approach where content can be downloaded and watched or shared offline will circumvent this problem (Madziva et al. 2022).

#### Government response to the infodemic

The *South African Disaster Management Act (DMA)* was promulgated in response to natural or climate-induced disasters. The DMA does not provide for human-made disasters such as an infodemic and a search of the document showed there is no mention of terms such as ‘infodemics’, ‘fake news’, ‘social media’ or ‘technology’. The DMA does not deal with how to mitigate an infodemic, but there is a reference to the national centre being a repository and conduit for disaster information (Kunguma 2021).

National governments should have a risk communication strategy in place for emergency situations, including public health emergencies such as COVID-19. The benefit of a risk communication strategy is that it can reduce the risk of spreading misinformation among citizens as the accurate information is available (Nannyonga et al. 2020). Part of the risk communication strategy will include how emergency communication will be established during the health crises. The strategy needs to have support from health partners, experts, communities and civil society as coordinated efforts from all these stakeholders will instil trust and confidence in citizens (Katurura & Cilliers 2018).

Risk communication must be frequent and allow for two-way feedback between all levels of government and citizens. The benefit of this is that information is available in real time and that resources can be allocated to the areas that need it the most based on data. However, risk communication is not enough to ensure social behaviour change but must be enforced by compliance activities and on-the-ground surveillance of adherence to preventative measures in public areas (Leburu et al. 2022).

The responsibility of the government extends further than just communication during a public health pandemic. To avoid pandemic fatigue, content must be innovative enough to captivate the audience as they receive repetitive information about the pandemic. Furthermore, market research needs to be conducted on preferred communication channels, mobile device penetration rates, access to data and traditional media channels (Leburu et al. 2022). These data can be collected during household visits, interactive radio shows, social media or WhatsApp (Erlach et al. 2021).

Adebisi et al. (2021b) found that most African countries (Kenya, Cameroon, Ghana, Tanzania, Nigeria, South Africa and Uganda) do have risk communication strategies in place that focus on training and capacity building, risk communication systems, internal and partners’ coordination, community engagement, public communication, contending uncertainty, addressing misperceptions and managing misinformation. Specific strategies that should be addressed in the risk communication plan include how information surveillance will be conducted, the dissemination of COVID-19 statistics and situation reports and what social media will be used during the public health crises (Adebisi, Rabe & Lucero-Prisno 2021a; Ahinkorah et al. 2020; Nannyonga et al. 2020). Effective risk communication has the advantage that it builds trust, credibility, honesty, transparency and accountability between the citizens of the country and the government (Atuguba & Atuguba 2020).

Social listening refers to the tracking, analysis and synthesis of community inputs both online and offline. It is one of the most important behavioural response strategies to identify rumours and fake news to respond effectively to misinformation (Sommariva et al. 2021). Sommariva et al. (2021) reported that Kenya, Comoros, Madagascar, Malawi and Zambia had all implemented various social listening tools to monitor the most prevalent questions, metrics and information about COVID-19 on social media platforms. The Africa Infodemic Response Alliance (AIRA) uses social media listening tools to implement social listening, which tracks misinformation and mitigates the effect by creating content with well-known and respected experts who disprove the rumours and myths (Ennab et al. 2022; Stewart et al. 2022). In addition, social media users have the responsibility to confirm the source before sharing information (Adekoya & Fasae 2021). Furthermore, users should engage in the whole article, not just the headlines that tend to be very sensationalist in nature (Olatunji et al. 2020).

### Verification mechanisms on social media

Misinformation is spread on social media without any intention to deceive others, the user is simply sharing information that they believe to be true or helpful to others (Adekoya & Fasae 2021). Unfortunately, fake news tends to spread faster on social media than accurate news, but there seems to be less engagement with fake news tweets when compared with science-based tweets on Twitter (Gbashi et al. 2021). This means that the information available on social media must be verified for accuracy. Fact-checking is important as misinformation is spread from news sources based abroad for example, Africa Check debunked a New York Times story that claimed South Africa had the fifth highest number of COVID-19 infections in the world (Ataguba & Ataguba 2020).

To improve the verification of COVID-19 information, Facebook has implemented a fact-checking pilot project while mass media agencies engage in pre- and post-fact-checking of news items (Adekoya & Fasae 2021; Etim, Iyamu & Chinwuba 2020; Olatunji et al. 2020). In 2020, Twitter updated its policy to include content sharing from authoritative sources of health information while Google put mechanisms in place that trigger an ‘SOS Alert’ when a search on COVID-19 is requested that gives preference to results from the WHO and the United States Centre for Disease Control and Prevention (Etim et al. 2020; Gbashi et al. 2021). Cooperation between various health agencies and social media platforms will further improve efforts to verify the information and thus prevent the spread of misinformation.

### Stakeholders to mitigate infodemics

In order to curb misinformation, various stakeholders need to collaborate and share their expertise in the problem area. Stakeholders in this process include social media platforms, health organisations, civil society, public authorities and figures, tech companies, traditional media and medical associations. The rest of the section will discuss the various role players that were identified during the COVID-19 pandemic and their role to fight the infodemic.

The WHO has offices in various countries that provide technical cooperation and leadership in the health sector. These country offices provide policy advice and technical support, public relations and advocacy and health management to countries (Chisita & Ngulube 2022).

Global Pulse is a United Nations collaboration that provides tools that monitor talks on radio, find influencers and identifies those who spread rumours (Ennab et al. 2022). The International Federation of Red Cross and Red Crescent societies (IFRC) provides feedback and complaints systems that could be used to systematically listen to communities and respond to citizens (Erlach et al. 2021). The IFRC published a 5-step process for community engagement with the objective to improve decision-making during public health emergencies (Lal et al. 2022).

The Africa Infodemic Response Alliance (AIRA) is a WHO partner organisation that started in 2020. The AIRA uses listening tools to identify and track fake news on social media and then produces scientifically accurate information to counter these claims. Viral Fact Africa is a social content hub that develops and circulates content that corrects COVID-related misinformation. Both these organisations work with public health organisations and fact checkers (Ennab et al. 2022). Media Monitoring Africa is a media organisation that promotes the development of a critical and ethical media culture in Africa. Media Monitoring Africa provides a website called Real411 where the public can report disinformation about COVID-19 (Gbashi et al. 2021). AfricaCheck.org is a verification site that fact-checks a variety of information, including COVID-19-related misinformation (Offer-Westort, Rosenzweig & Athey 2021). AfricaCheck.org operates in South Africa, Kenya and Nigeria while there is a similar organisation operating in Zimbabwe and Namibia. The mission of AfricaCheck.org is to help the media to deliver accurate, fair and balanced news and information (Mare & Munoriyarwa 2022).

Locally, libraries have been identified as a source of quality and credible COVID-19 resources and information on their websites and Library Guides. The Universities of Stellenbosch, Pretoria, and Cape Town were the first to provide current information about COVID-19 on their library websites while most other universities provided links to free COVID-19 resources on their homepages as per government regulations (Bangani 2021; Chisita 2020).

**FIGURE 2:**
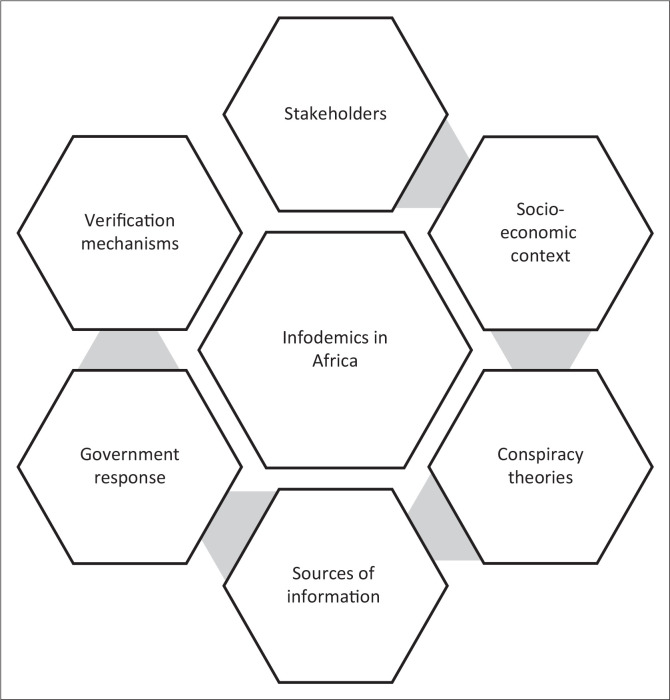
Summary of factors that impact infodemics in Africa.

Urban observatories collect, analyse and present urban data to enable factual decision-making for policymakers. An example is the Gauteng City-Region Observatory (GCRO), which was established in 2008 and operates in Johannesburg, South Africa. The GCRO works in partnership with the University of Johannesburg and the University of the Witwatersrand. During the COVID-19 pandemic, GCRO provided a crisis advisory role through data visualisation and analytics capacity that allowed the local government to interpret the evolving situation in Gauteng in terms of COVID-19 vulnerabilities (Acuto et al. 2021; Bangani 2021). Figure 2 provides a summary of the factors that were discussed in the preceding sections.

## Conclusion and recommendations

The purpose of this strategic review of recently published and relevant literature was to describe the available research concerning the role of social media platforms in creating and reinforcing an infodemic during health pandemics in Africa. With the increase in infodemics as realised through the COVID-19 pandemic and other preceding epidemics and endemics, it is evident that social media has been the leading channel for the spread of misinformation. However, in Africa, there has been notable COVID-19 misinformation in rural areas where there is limited or no social media usage.

The study identified six factors that impact infodemics in Africa. These included stakeholders, socio-economic context, conspiracy theories, sources of information, government responses and verification mechanisms. The findings of this study indicate that there are various stakeholders that must be considered during an infodemic. The government also needs to include infodemics in the risk communication strategy for public health emergencies. Verification of misinformation can mitigate the effects of conspiracy theories while the socio-economic context of the audience must be taken into consideration when planning strategies to mitigate infodemics on social media.

The contribution of the study is in the field of risk communication during pandemics on the African continent. The six themes that were identified contributes to a more effective response during pandemics and assist various stakeholders on how to prepare for such events in the future. The study also contributes to the research agenda in the field of infodemics by producing an evidence-based response to the pandemic in Africa.

## Appendix

**TABLE 1-A1:** Articles used for the structured literature review.

Article title		Year published	African country/countries covered	Pandemic, epidemic or endemic covered	Media investigated (even in literature review discussion)	Research methodology	General area(s) of study
1	Mare, A. & Munoriyarwa, A., 2022, ‘Guardians of truth? Fact-checking the “disinfodemic” in Southern Africa during the COVID-19 pandemic’, *Journal of African Media Studies* 14(1), 63–79.	2022	Namibia, South Africa and Zimbabwe	COVID-19	Social media, WhatsApp, Twitter	Qualitative – Virtual ethnography and online interviews	Disinfodemic, misinformation, disinformation, fact checking
2	Ennab, F., Babar, M.S., Khan, A.R., Mittal, R.J., Nawaz, F.A., Essar, M.Y. & Fazel, S.S., 2022, ‘Implications of social media misinformation on COVID-19 vaccine confidence among pregnant women in Africa’, *Clinical Epidemiology and Global Health* 14, 100981.	2022	Africa	COVID-19	Social media	Qualitative	Misinformation, infodemic
3	Leburu, N., Shilumani, C., Bhengu, C., Matlala, N., Maja, P., Jimoh, S. et al., 2022, ‘Risk communication and community engagement–unlocking the key to South Africa’s response to SARS-CoV-2’, *South African Medical Journal* 112(5b), 366.	2022	South Africa	COVID-19	Digitally published documents, social media, traditional media	Qualitative –review of documents	Infodemic, Misinformation, risk communication, community engagement
4	Madziva, R., Nachipo, B., Musuka, G., Chitungo, I., Murewanhema, G., Phiri, B. et al., 2022, ‘The role of social media during the COVID-19 pandemic: Salvaging its ‘power’ for positive social behaviour change in Africa’, *Health Promot* 12(1), 23.	2022	Africa, Zimbabwe	COVID-19	Social media	Qualitative	Infodemic, communication for development (C4D)
5	Lal, A., Ashworth, H.C., Dada, S., Hoemeke, L. & Tambo, E., 2022, ‘Optimising pandemic preparedness and response through health information systems: Lessons learned from Ebola to COVID-19’, *Disaster Medicine and Public Health Preparedness* 16(1), 333–340.	2022	West Africa, DRC	Ebola, COVID-19	Social media	Qualitative	Infodemic, misinformation, community engagement, response and preparedness
6	Stewart, R., Madonsela, A., Tshabalala, N., Etale, L. & Theunissen, N., 2022, ‘The importance of social media users’ responses in tackling digital COVID-19 misinformation in Africa’, *Digital Health* 8, 20552076221085070.	2022	Africa (17 countries)	COVID-19	Social media	Multi-method – rapid review research and a survey via WhatsApp	Misinformation, communication, digital technologies
7	Ajekwe, P.O., 2022, ‘The impact of social media on Covid-19 information management system: A review of literature’, *ANSU Journal of Arts and Social Sciences (ANSUJASS)* 9(1), 45–57.	2022	Nigeria	COVID-19	Social media	Qualitative	Infodemic, social media
8	Chisita, C.T. & Ngulube, P., 2022, ‘A framework for librarians to inform the citizenry during disasters: Reflections on the COVID-19 pandemic’, *Jàmbá-Journal of Disaster Risk Studies* 14(1), 1197.	2022	Southern Africa, Malawi	COVID-19	Internet, social media	Qualitative – QCA	Infodemic, coroinfodulgence, disinformation
9	Gagliardone, I., Diepeveen, S., Findlay, K., Olaniran, S., Pohjonen, M. & Tallam, E., 2021, ‘Demystifying the COVID-19 infodemic: Conspiracies, context, and the agency of users’, *Social Media+ Society* 7(3), 20563051211044233.	2021	South Africa, Nigeria	COVID-19	Twitter	Mixed method analysis	COVID-19 infodemic, conspiracy theories, social media, mis/disinformation
10	Okereke, M., Ukor, N.A., Ngaruiya, L.M., Mwansa, C., Alhaj, S.M., Ogunkola, I.O. et al., 2021, ‘COVID-19 misinformation and infodemic in≈rural Africa’, *The American Journal of Tropical Medicine and Hygiene* 104(2), 453.	2021	Rural Africa (Sudan, Kenya, Tanzania, Nigeria, Egypt)	COVID-19	Lack of access to information, including social media channels	Qualitative	Misinformation, infodemic
11	Adekoya, C.O. & Fasae, J.K., 2021, ‘Social media and the spread of COVID-19 infodemic’, *Global Knowledge, Memory and Communication* 71(3), 105–120.	2021	Nigeria	COVID-19	Social media applications (WhatsApp, Zoom)	Quantitative – descriptive survey	Fake news, infodemic
12	Sommariva, S., Mote, J., Ballester Bon, H., Razafindraibe, H., Ratovozanany, D., Rasoamanana, V. et al., 2021, ‘Social listening in Eastern and Southern Africa, a UNICEF risk communication and community engagement strategy to address the COVID-19 infodemic’, *Health Security* 19(1), 57–64.	2021	Eastern and Southern Africa (Comoros, Kenya, Madagascar, Malawi, and Zambia)	COVID-19	Online platforms, social media and instant messaging apps	Qualitative – descriptive study	Infodemic, risk communication, social listening
13	Manda, L.Z., 2021, ‘Exploring COVID-19 infodemic in rural Africa: A case study of Chintheche, Malawi’, *Journal of African Media Studies* 13(2), 253–267.	2021	Malawi	COVID-19	Facebook, Twitter, WhatsApp	Qualitative –Interviews	Disinformation, infodemic, technological denialist infodemics
14	Bangani, S., 2021, ‘The fake news wave: Academic libraries’ battle against misinformation during COVID-19’, *The Journal of Academic Librarianship* 47(5), 102390.	2021	South Africa	COVID-19	Facebook, Twitter	Qualitative	Fake news, infodemic, information literacy
15	Demuyakor, J., Nyatuame, I.N. & Obiri, S., 2021, ‘Unmasking covid-19 vaccine “infodemic” in the social media’, *Online Journal of Communication and Media Technologies* 11(4), e202119.	2021	Ghana	COVID-19	Social media	Quantitative – online survey	Infodemic, trust/mistrust/distrust
16	Gbashi, S., Adebo, O.A., Doorsamy, W. & Njobeh, P.B., 2021, ‘Systematic delineation of media polarity on COVID-19 vaccines in Africa: Computational linguistic modeling study’, *JMIR Medical Informatics* 9(3), e22916.	2021	Africa	COVID-19	Twitter, Google News headlines	Quantitative	Infodemic, sentiment
17	Dzinamarira, T., Nachipo, B., Phiri, B. & Musuka, G., 2021, ‘COVID-19 vaccine roll-out in South Africa and Zimbabwe: Urgent need to address community preparedness, fears and hesitancy’, *Vaccines* 9(3), 250.	2021	South Africa, Zimbabwe	COVID-19	Internet, social media	Qualitative	Misinformation, vaccine hesitancy
18	Offer-Westort, M., Rosenzweig, L.R. & Athey, S., 2021, *Optimal policies to battle the coronavirus “infodemic” among social media users in Sub-Saharan Africa*, OSF Registered Study.	2021	Sub-Saharan Africa (Kenya and Nigeria)	COVID-19	Facebook Messenger	Quantitative	Infodemic, misinformation, social media, policies
19	Kunguma, O., 2021, ‘COVID-19 home remedies and myths becoming a hazardous health infodemic?’, *Jàmbá: Journal of Disaster Risk Studies* 13(1), 1–4.	2021	South Africa	COVID-19	*Disaster Management Act* digital media articles	Qualitative – exploratory study	Infodemics, public health, home remedies
20	Shobowale, O., 2021, ‘A systematic review of the spread of information during pandemics: A case of the 2020 COVID-19 virus’, *Journal of African Media Studies* 13(2), 221–234.	2021	Africa	COVID-19; Ebola and HIV	Internet sources of academic publications – Google Scholar, PubMed, MEDLINE, Nexus Uni, Wiley Online Library, Frontiers Media, Nature and ScienceDirect	Qualitative - Systematic review	Infodemic, misinformation, fake news
21	Osuagwu, U.L., Miner, C.A., Bhattarai, D., Mashige, K.P., Oloruntoba, R., Abu, E.K. et al., 2021, ‘Misinformation about COVID-19 in sub-Saharan Africa: Evidence from a cross-sectional survey’, *Health Security* 19(1), 44–56.	2021	Saharan Africa (Cameroon Ghana, Kenya, Nigeria, South Africa, Tanzania, and Uganda)	COVID-19 Ebola and HIV	Facebook and WhatsApp	Quantitative- Cross sectional survey	Misinformation, infodemic
22	Acuto, M., Dickey, A., Butcher, S. & Washbourne, C.L., 2021, ‘Mobilising urban knowledge in an infodemic: Urban observatories, sustainable development and the COVID-19 crisis’, *World Development* 140, 105295.	2021	Johannesburg, Karachi, Freetown and Bangalore	COVID-19	Digital media from urban observatories	Qualitative – case studies	Infodemic, urban observatories
23	Ademola, S.S., Rajabu, N., Umezuruike, C. & Alamu, L.K., 2021, ‘Effect of COVID-19 infodemic on media trust and perceived stress’, *Journal of Health Promotion and Behavior* 6(02), 143–152.	2021	Africa	COVID-19	Twitter	Quantitative – Cross sectional study	Infodemic, health communication, media trust
24	Chimoyi, L., Mabuto, T., Dube, T., Ntombela, N., Nchachi, T., Tshisebe, D. et al., 2021, ‘The geography of COVID-19 misinformation: Using geospatial maps for targeted messaging to combat misinformation on COVID-19, South Africa’, *BMC Research Notes* 14(1), 1–6.	2021	South Africa	COVID-19	Social media	Quantitative	Infodemic, misinformation, GIS
25	Adebisi, Y.A., Rabe, A. & Lucero-Prisno III, D.E., 2021, ‘Risk communication and community engagement strategies for COVID-19 in 13 African countries’, *Health Promotion Perspectives* 11(2), 137.	2021	Algeria, Ghana, South Africa, Tanzania, Kenya, Mauritius, Angola, Cote d’Ivoire, Ethiopia, the Democratic Republic of the Congo, Nigeria, Zambia, and Uganda	COVID-19	Social media platforms	Qualitative – narrative review	Risk communication, community engagement
26	Erlach, E., Nichol, B., Reader, S. & Baggio, O., 2021, ‚Using community feedback to guide the COVID-19 response in sub-Saharan Africa: Red Cross and Red Crescent approach and lessons learned from Ebola’, *Health Security* 19(1), 13–20.	2021	Sub-Saharan Africa	COVID-19, Ebola	Social media	Qualitative	Risk communication, community engagement, infodemic
27	Wasserman, H., Chuma, W., Bosch, T., Uzuegbunam, C.E. & Flynn, R., 2021, ‘South African newspaper coverage of COVID-19: A content analysis’, *Journal of African Media Studies* 13(3), 333–350.	2021	South Africa	COVID-19	Newspaper publications	Quantitative – QCA	Infodemic, misinformation, media framing
28	Aduloju, E.T., 2021, Media and information literacy: A critical response to the challenge of ‘Infodemic’in the Covid-19 pandemic era in Nigeria’, *Resisting Disinfodemic Media and Information Literacy* 80.	2021	Nigeria	COVID-19	Social media	Qualitative – textual analysis	Infodemic, misinformation, information literacy
29	Adebisi, Y.A., Rabe, A. & Lucero-Prisno III, D.E., 2021, ‘COVID-19 surveillance systems in African countries’, *Health Promotion Perspectives* 11(4), 382.	2021	Mauritius, Algeria, Nigeria, Angola, Cote d’Ivoire, the Democratic Republic of the Congo, Ghana, Ethiopia, South Africa, Kenya, Zambia, Tanzania, and Uganda	COVID-19	Social media	Qualitative – narrative review	Surveillance, misinformation
30	Aiyebelehin, A.J. & Mesagan, F.O., 2021, ‚Mitigating the infodemic associated with the COVID-19 pandemic: Roles of Nigerian librarians’, *IAFOR Journal of Literature and Librarianship* 62–75.	2021	Nigeria	COVID-19	Social media	Quantitative	Infodemic, social media, fake news
31	Schmidt, T., Cloete, A., Davids, A., Makola, L., Zondi, N. & Jantjies, M., 2020, ‘Myths, misconceptions, othering and stigmatizing responses to Covid-19 in South Africa: A rapid qualitative assessment’, *PLoS One* 15(12), e0244420.	2020	South Africa	COVID-19, HIV	Social media	Qualitative	Misconceptions
32	Ogola, G., 2020, ‘Africa and the Covid-19 information framing crisis’, *Media and Communication* 8(2), 440–443.	2020	Africa	COVID-19	Social media	Qualitative	Misinformation, news framing
33	Ataguba, O.A. & Ataguba, J.E., 2020, ‘Social determinants of health: The role of effective communication in the COVID-19 pandemic in developing countries’, *Global Health Action* 13(1), 1788263.	2020	South Africa	COVID-19	Social media	Qualitative	Risk communication, effective communication, developing countries
34	Ahinkorah, B.O., Ameyaw, E.K., Hagan, Jr, J.E., Seidu, A.A. & Schack, T., 2020, ‘Rising above misinformation or fake news in Africa: Another strategy to control COVID-19 spread’, *Frontiers in Communication* 5, 45.	2020	Africa	COVID-19	Social media	Qualitative	Misinformation, fake news, mass media
35	Nannyonga, B.K., Wanyeze, R.K., Kaleebu, P., Ssenkusu, J. M., Ssengooba, F., Lutalo, T. et al., 2020**,** *Infodemic: How an epidemic of misinformation could lead to a high number of the novel corona virus disease cases in Uganda*, pp. 1–8.	2020	Uganda	COVID-19	Social media	Quantitative	Misinformation, infodemic
36	Etim, G.L., Iyamu, I. & Chinwuba, C.C., ‘Analytical approach to ascertain the origin of different kinds of news on corona virus (Covid-19) pandemic across social media’, *International Journal of Innovative Science and Research Technology* (5)5, 292–296.	2020	Nigeria	COVID-19	Social media, online news	Qualitative	Infodemic, social media, fake news
37	Lucas, J.M., Targema, T.S., Jibril, A., Sambo, E.O. & Istifanus, B.A., 2020, ‘Combating COVID-19 infodemic in Nigerian rural communities: The imperatives of Traditional Communication Systems’, *ASEAN Journal of Community Engagement* 4(2), 360–385.	2020	Nigeria	COVID-19	Modern media platforms – social media, websites	Qualitative – generate data from secondary sources – books, journal articles, corporate websites, technical reports, newspaper and media reports, and databases of agencies	Infodemic, rural areas, traditional communication systems
38	Chisita, C.T., 2020, ‘Libraries in the midst of the Coronavirus (COVID-19): Researchers experiences in dealing with the vexatious infodemic’, *Library Hi Tech News* 37(6), 11–14.	2020	Africa	COVID-19	Library databases – Web of Science, Scorpius, PubMed, Eric, JSTOR, ScienceDirect, Directory of Open Access Journals, etc. Google Scholar	Qualitative	Infodemic, information disorder, Covidinfo-deluge
39	Olatunji, O.S., Ayandele, O., Ashirudeen, D. & Olaniru, O.S., 2020, ‘“Infodemic” in a pandemic: COVID-19 conspiracy theories in an African country’, *Social Health and Behavior* 3(4), 152.	2020	Nigeria	COVID-19	Social media, the internet, mass media	Quantitative – cross sectional study	Conspiracy theories, infodemic
40	Ufearoh, A.U., 2020, ‘COVID-19 pandemic as an existential problem: An African perspective’, *Filosofia Theoretica: Journal of African Philosophy, Culture and Religions* 9(1), 97–112.	2020	Africa	COVID-19	Social media	Qualitative	Infodemics, virus of disinformation, anthropology
41	Balami, A.D. & Meleh, H.U., 2019, ‘Misinformation on salt water use among Nigerians during 2014 Ebola outbreak and the role of social media’, *Asian Pacific Journal of Tropical Medicine* 12(4), 175.	2019	Nigeria	Ebola	Social media	Quantitative	Misinformation
